# HLA class I expression shapes the tumor immune microenvironment and influences prognosis in prostate cancer

**DOI:** 10.1038/s41391-025-01045-9

**Published:** 2025-10-25

**Authors:** Pornlada Likasitwatanakul, Carissa Besonen, Alexander K. Tsai, Negar Sadeghipour, Andrew Elliott, Ali T. Arafa, Rachel Passow, Lisa Chesner, Martin Felices, Philippa R. Kennedy, Akash Patnaik, Vivek Narayan, James Hamrick, Laura A. Sena, Nicholas A. Zorko, Justin H. Hwang, Emmanuel S. Antonarakis

**Affiliations:** 1 Department of Medicine, University of Minnesota-Twin Cities, Minneapolis, MN 55455 USA; 2 Department of Medicine, Siriraj Hospital, Mahidol University, Bangkok, 10700 Thailand; 3 School of Medicine, Creighton University, Omaha, NE 68178 USA; 4 Masonic Cancer Center, University of Minnesota-Twin Cities, Minneapolis, MN 55455 USA; 5 Department of Medical Affairs, Caris Life Sciences, Irving, TX 85040 USA; 6 Department of Urology, University of California San Francisco, San Francisco, CA 94115 USA; 7 Section of Hematology/Oncology, Department of Medicine, University of Chicago, Chicago, IL 60637 USA; 8 Department of Medicine, University of Pennsylvania, Philadelphia, PA 19104 USA; 9 Sidney Kimmel Comprehensive Cancer Center, Johns Hopkins University, Baltimore, MD 21231 USA

**Keywords:** Translational research, Prognostic markers, Cancer genetics, Prostate cancer

## Abstract

**Background:**

Human leukocyte antigen (HLA) class I encompasses peptide-binding proteins that regulate T-cell interactions. We examined HLA class I expression in prostate cancers (PC), exploring associations with clinical outcomes, molecular features, and tumor immune microenvironment.

**Methods:**

We analyzed 8040 PC samples from the Caris Life Sciences database, stratifying them into HLA-high (upper quartile) and -low (lower quartile) groups. Genomic and transcriptomic alterations were compared. Immune cell fractions were inferred using quanTIseq, and overall survival (OS) data was obtained from insurance claims. Differences were computed with Cox proportional hazards.

**Results:**

Among 66 cancer types, PC ranked 3^rd^-, 11^th^-, and 19^th^-lowest for HLA-A, -B, and -C expression, respectively. In PC, genes tied to androgen receptor (*AR*) signaling, immune checkpoint molecules (*CTLA4, PD-L1*), and the epithelial-mesenchymal transition were significantly higher in HLA-high tumors. HLA-high status was linked to greater tumor immune activity, marked by higher T cell fractions and enhanced immune hallmarks. HLA-high tumors were less likely to possess alterations in *AR, FOXA1*, and *CDK12*, but harbored increased alterations in tumor suppressor gene (*RB1*, *PTEN*) alterations. Tumors with high HLA-A and HLA-B had elevated TMB-H/MSI-H/dMMR status. Finally, shorter OS was observed in patients with high HLA-A or HLA-B expression, while longer OS was associated with high HLA-C expression.

**Conclusions:**

In PC, elevated HLA class I levels correlate with immune activity, molecular characteristics, and clinical outcomes. We suggest considering HLA expression as a supplementary marker of immune activity in PC, alongside genetic mutations and transcriptomic markers.

## Introduction

Prostate cancer (PC) is the most commonly diagnosed malignancy in men globally [1, 2]. The androgen receptor (AR) is the main therapeutic target for the current standard treatment of PC [3, 4]. However, resistance to androgen deprivation therapy (ADT) ultimately leads to cancer progression and a need for other treatment options [5]. ADT resistance is associated with changes in the tumor microenvironment (TME), which impacts tumor immune cell interactions and responses to immune therapies against PCs [6, 7]. Altogether, it is essential to examine other factors shaping the TME to identify other avenues for targeted therapeutics and to inform PC prognosis.

Class I human leukocyte antigens (HLA) comprise HLA-A, HLA-B, and HLA-C subclasses [8]. These encode peptide-binding domains on the cell surface and mediate T cell activation, thereby directing cytotoxic T cell-based antitumor immunity. HLA downregulation on cancer cells is an established mechanism of immune escape. As evidence of this, HLA allele deletion, or loss of heterozygosity, is more frequently observed in advanced cancers relative to primary tumors and is associated with poor prognosis and limited response to immune checkpoint inhibitors (ICI) in some malignancies [9–12]. PC, as compared to other cancers, is considered “immune-cold” with respect to tumor-infiltrating lymphocytes (TILs) and responses to ICIs [13], with <5% of PCs harboring markers of immunotherapy responsiveness [10–13]. Our recent case report of two PC nodules within the same patient demonstrated that despite both tumors being TMB-high and MMR-deficient divergent, HLA expression patterns can drive differences in immune cell interactions [14]. Separately, recent studies have indicated that HLA expression may be suppressed by AR signaling, in which AR blockade results in gaining responsiveness to immunotherapies [15, 16]. This evidence suggests that in prostate tumor cells, HLAs interact with various tumoral features that altogether mediate tumor and immune cell interactions.

This study is centered on the hypothesis that differences in classical HLA class I expression in PC will be associated with differences in the tumor microenvironment, somatic features, and clinical outcomes. By analyzing a database of molecularly-profiled real-world patient samples, we retrospectively examined 8,040 PC samples partitioned by high and low expression of HLA-A, -B, and -C. Our study is the first to analyze HLA class I allele expression using a large clinical cohort compared to previous studies (Supplementary Table 1). We identified differences in molecular features, signaling pathways, and patient outcomes, which suggests that consideration of HLA expression status may inform prognosis and facilitate future studies to improve outcomes for patients with PC.

## Methods

### Study approval

This study was conducted in accordance with the guidelines of the Declaration of Helsinki, Belmont report, and U.S. Common Rule. In keeping with 45 CFR 46.101(b) 32, this study was performed using retrospective, de-identified clinical data. Therefore, this study is considered institutional review board (IRB) exempt, and no patient consent was necessary from the subjects. This study has been granted an IRB exemption (Study No. 00013548). However, the construction of the source database and its use for research purposes have received IRB approval (IRB No. 00013548).

### Clinical information and survival analysis

We queried a de-identified dataset of real-world patients’ samples that underwent comprehensive molecular profiling at a CLIA/CAP-certified lab (Caris Life Sciences). Overall survival (OS) was defined as the date of tissue collection to either death or last contact extracted from insurance claims data. Hazard ratios (HR) were calculated using the Cox proportional hazards model, and P values were calculated using the log-rank test. Treatment information was derived from insurance-claimed data. Multivariate analyses were conducted adjusting for age, race, histology, *TP53*, TMB, and MSI statuses.

### Next-generation sequencing of the DNA

NGS was performed on genomic DNA isolated from formalin-fixed paraffin-embedded (FFPE) tumor samples using the NextSeq or NovaSeq 6000 platforms. Clinically relevant genes (either 592 or 700 genes) were sequenced at high coverage with an average sequencing depth of >500. Genetic variants identified were interpreted by board-certified molecular geneticists and categorized according to the American College of Medical Genetics and Genomics (ACMG) standards. ‘Pathogenic’ and ‘likely pathogenic’ were counted as mutations. The copy number alteration (CNA) of each exon is determined by calculating the average depth of the sample along with the sequencing depth of each exon and comparing this calculated result to a pre-calibrated value.

### HLA genotypes

The Caris WES HLA assay provides a genotype for the MHC Class I HLAs (A, B and C genes) down to the allele using OptiType [17], when reads are >10x depth. Samples with homozygous HLA were defined as having identical high-resolution HLA genotypes [18], while those with heterozygous HLA had different HLA genotypes. The RNA-Seq data is read by arcasHLA [19] to provide a genotype for the HLA alleles present and expressed in the patient sample. At least 100 reads for a given HLA Class I gene is necessary to obtain an HLA call for that gene via WTS.

### RNA expression

The same FFPE specimens were processed as previously discussed to collect RNA [20, 21]. The Illumina NovaSeq 6500 was then used to sequence the whole transcriptome from patients to an average of 60 M reads. Raw data was demultiplexed by Illumina Dragen BioIT accelerator, trimmed, and counted, and PCR-duplicates were removed and aligned to human reference genome hg19 by STAR aligner [22]. For transcription counting, transcripts per million values noted in transcripts per million (TPM) were generated using the Salmon expression pipeline [23].

### Transcription analysis

The Hallmarks of gene sets of Gene Set Enrichment Analysis was used to evaluate pathway enrichment [24]. Immune cell tumor infiltration was inferred using quanTIseq based on the deconvolution of bulk RNA-seq data as previously described [25].

### Statistical analysis

Continuous variables were compared using non-parametric tests, including Mann Whitney-U tests, while categorical data were compared using Chi-square or Fisher’s exact test, where appropriate. Comparisons among groups of more than three were made using the ANOVA test. Differential gene expression analysis was done using the Mann-Whitney U test and corrected for multiple comparisons using the Benjamini-Hochberg ad hoc test. A *p*-value of <0.05 or an adjusted p-value of < 0.05 was considered a significant difference [26]. Unless otherwise specified, in figures, the bars show min to max values, and asterisks represent the following *p*-values: * <0.05, ** <0.01, *** < 0.001. If there was no significant difference between the analysis (*p* > 0.05), the statistical significance was not shown on the graphs.

## Results

### Prostate cancers exhibit reduced HLA class I expression

**Fig. 1:**
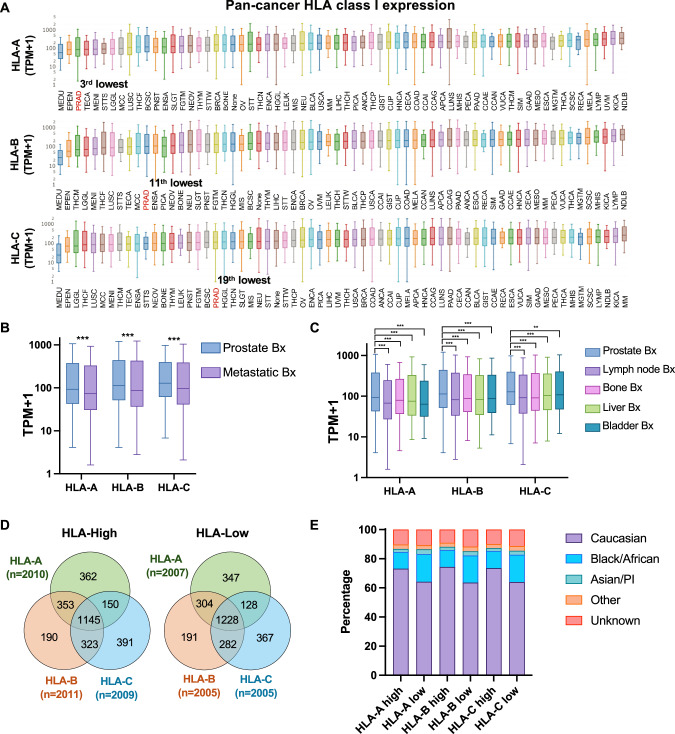
HLA class I expression pattern. . HLA class I expression among all cancers from the Caris database ranked by median expression level. Red text denotes prostate adenocarcinoma **A**. HLA class I expression among PC stratified by biopsy (bx) sites (primary prostate vs. metastatic sites) **B** and stratified by common metastatic sites **C**. The Venn diagram showed the number of PCs in each subgroup stratified by HLA class I expression **D**. Stacked bar graphs showing percentage of each race in each HLA expression subgroup **E**.

We investigated the Caris database, comprised of 10,759 PC samples, including 6,344 primary and 4,415 metastatic biopsies. Among these, HLA expression data was available for 75% (8,040) of samples (Supplementary Tables 2-3). Reported in TPM for all samples, we found that among 66 cancer types, PC ranked 3^rd-^, 11^th-,^ and 19^th-^lowest with respect to HLA-A, HLA-B, and HLA-C expression, respectively (Fig. 1A and Supplementary Table 4). When stratified by biopsy site, median expressions for all HLA class I loci were lower in biopsies obtained from metastatic sites as compared to primary sites (Fig. 1B). Among the most common metastatic sites, lymph node biopsies had the lowest HLA expression, followed by bone, liver, and bladder biopsies (Fig. 1C). Altogether, HLA class I expression in PC was low compared to other cancers, with expression levels decreased further in metastatic tumors relative to primary tumors.

To explore the impact of HLA class I expression in PC, we stratified PC samples into HLA-high (>75th percentile; upper quartile) and -low (<25th percentile; lower quartile) groups for HLA-A, HLA-B, and HLA-C expression (Fig. 1D). When examining these HLA-high and -low groups with respect to self-reported race, white PC patients had significant increases in the HLA-high group, whereas African Americans demonstrated the opposite trend **(**Fig. 1E and Supplementary Table 5**)**. PCs appear to regulate the expression of all three HLA class I genes in a concordant fashion. However, our results also indicate that racial background influences HLA expression.

### High HLA expression is associated with increased alterations of tumor suppressor genes and decreased AR-related mutations

**Fig. 2:**
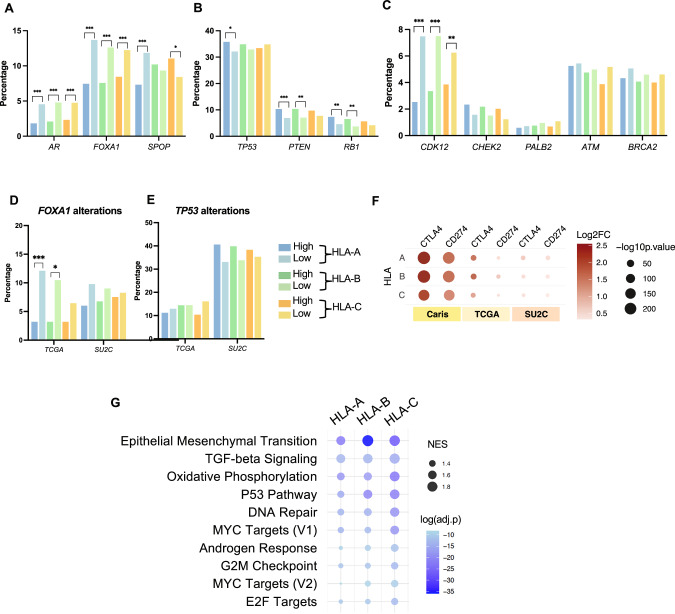
Genomic and transcriptomic correlation. Bar graphs showing percentages of pathogenic alterations in AR-related genes **A**, tumor suppressors **B**, and HRR-pathway genes **C**. Bar graphs showing percentages of pathogenic alteration of *FOXA1* **D** and *TP53* **E** in TCGA and SU2C cohorts. Color key for figure **A**–**E** is presented next to the figure **E**. Bubble plots showing differential gene expression of *CTLA4* and *CD274* (*PD-L1*) in the Caris cohort, compared to the TCGA and SU2C cohorts **F**. Bubble plots showing gene set enrichment analysis (GSEA) of cancer-related hallmark gene sets relative to HLA-high groups **G**.

To investigate the molecular associations of increased HLA class I expression, we compared the prevalence of pathogenic alterations between HLA-high and -low subgroups. Pathogenic alterations in *AR* and *FOXA1*, a pioneering AR transcription co-factor [27, 28], were more common in the HLA-low relative to the HLA-high groups by two-fold. Alterations of *SPOP*, another regulator of AR protein stability [29], was significantly higher in the HLA-A–low group but lower in the HLA-C–low group. (Fig. 2A). We next examined tumor suppressors that drive poor clinical outcomes in PC [30–33]. HLA-A and HLA-B–high groups had elevated rates of *PTEN* and *RB1* alterations, while only the HLA-A–high group had elevated *TP53* alterations (Fig. 2B). PCs harboring mutations in homologous recombination repair (HRR) genes can be managed with PARP inhibitors [34], therefore we also explored HRR gene alterations. Interestingly, PC with high HLA expression had reduced *CDK12* mutations, but no differences in other HRR genes (Fig. 2C). Some of the genomic findings were validated in two external cohorts: primary prostate cancer from the Cancer Genome Atlas Program (TCGA) and advanced prostate cancer from the Stand Up To Cancer (SU2C), especially for *FOXA1* and *TP53* alterations (Fig. 2D, E and Supplementary Fig. 1A, B). In summary, elevated expression of HLA was associated with numerous somatic characteristics that regulate PC progression, including key regulators that may activate AR signaling and alterations in tumor suppressor genes. Conversely, *CDK12* mutations may drive low HLA expression.

### Increased HLA expression is associated with elevated PD-L1 and CTLA4 expression and epithelial-mesenchymal transition (EMT)

Next, we examined transcriptomes to determine the association of HLA expression with genes or pathways that regulate PC progression. HLA-high vs. -low tumors harbored significant transcriptomic differences. Genes involved in AR regulation, immunoglobins, immune targets, and neuroendocrine differentiation [14, 31, 35] were generally enriched in HLA-high patients (Supplementary Fig. 2 and Supplementary Table 6–9). We then examined targets of immunotherapy and cell surface proteins that represent therapeutic targets in PC [36–40] (Supplementary Fig. 3A**)**. *CD274* (*PD-L1*) and *CTLA4* were significantly enriched in all HLA-high groups, which were consistent in 2 external cohorts (Fig. 2F). *AR*- and NEPC-related genes were variably expressed in different cohorts (Supplementary Fig. 3A–C). To identify biological pathways associated with HLA expression, we performed a GSEA study. Among Hallmark pathways, EMT, TGF-β signaling, and oxidative phosphorylation were highly enriched (NES > 1.5) in all HLA class I–high groups, while *TP53* and DNA repair were enriched in HLA-B and HLA-C–high groups (Fig. 2G). Altogether, increased HLA class I expression was associated with somatic alterations of genes in the AR pathway, as well as druggable cell surface proteins and pro-tumor signaling pathways.

### HLA expression is associated with increased T cells and immunotherapy markers

**Fig. 3:**
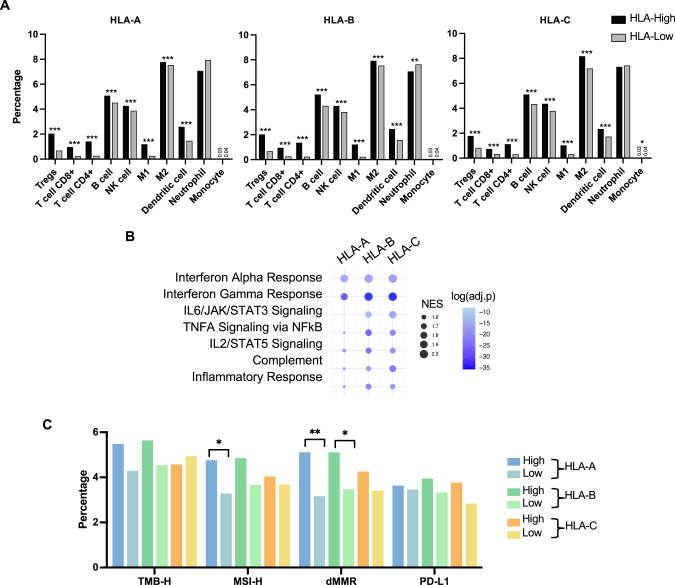
Tumor micro-environment differences. Bar graphs showing mean percentages of immune cells inferred by quanTIseq between PC with high and low HLA class I expression **A**. Bubble plots showing GSEA of hallmark gene sets related to immunologic processes relative to HLA-high groups **B**. Bar graphs showing percentages of immunotherapy-related markers (TMB-high, MSI-high, MMR deficient, and PD-L1 positive) **C**.

We next examined the association of HLA expression with immune infiltrates using quanTIseq [25]. Regulatory T cells (Tregs), cytotoxic T cells (CD8 + ), and helper T cells (CD4 + ) were all significantly increased across HLA-high groups (Fig. 3A). B cells, NK cells, M1 and M2 macrophages, and myeloid dendritic cells were all increased in HLA-high groups. IFNα and IFNγ pathways were highly enriched (NES > 1.8) in all HLA-high groups. Other pathways, including IL2, IL6, TNF-α, complement, and inflammatory response, were also generally enriched (NES > 1.5) in HLA-high groups, particularly in HLA-B and HLA-C loci (Fig. 3B). HLA-A and HLA-B–high tumors exhibited numerical increases in microsatellite instability (MSI-H) and deficiencies in mismatch repair (dMMR) status (Fig. 3C). These analyses indicate that elevated HLA expression is associated with increased T cell infiltration, augmentation of various immune regulatory pathways, as well as enrichment of ICI biomarkers.

### Genomic mechanisms of HLA expression

**Fig. 4:**
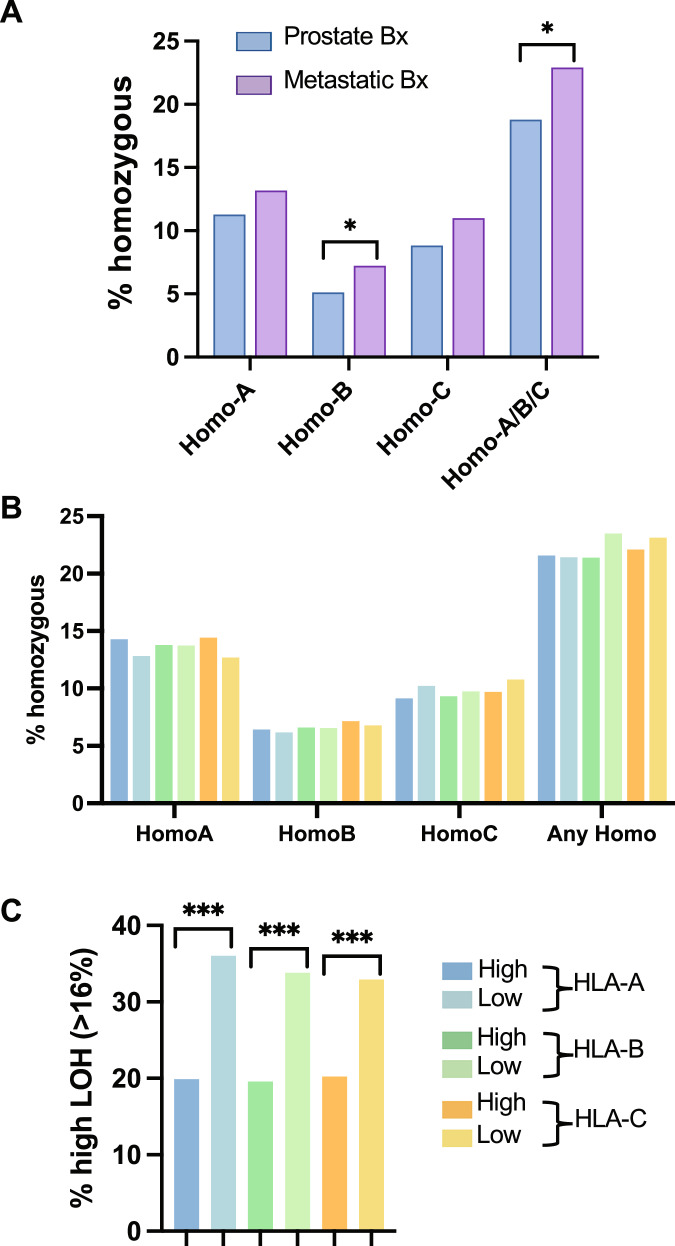
HLA class I zygosity and genome-wide LOH. Bar graphs showing the percentage of PC with HLA homozygosity in primary prostate and metastatic biopsies (Bx) **A**. Bar graphs showing the percentage of PC with HLA homozygosity **B** and PC with high genome-wide LOH ( > 16%) **C** stratified by HLA-high and HLA-low groups. Color key for figure **B**, **C** is presented next to the figure **C**.

To understand the mechanisms of differential HLA expression and potential relationships with allele zygosity, we explored associations between HLA zygosity and HLA expression. Compared to primary tumors, metastatic tumors exhibited a higher proportion of samples that were homozygous for HLA-B and homozygous for any of HLA-A/B/C (Fig. 4A). However, when considering all PC samples together, the HLA-high and -low groups did not exhibit differences in the rates of HLA homozygosity (Fig. 4B). Genome-wide loss of heterozygosity (gLOH) is another potential mechanism that may influence HLA expression [41, 42]. PC with low HLA expression exhibited a ~ 1.7-fold greater prevalence of high gLOH (Fig. 4C). Altogether, in PC, there may be complex mechanisms that regulate HLA expression beyond allele homozygosity and genome-wide LOH status. Furthermore, a greater degree of gLOH may result in an adaptive suppression of HLA class I expression.

### HLA class I expression and survival outcomes

**Fig. 5:**
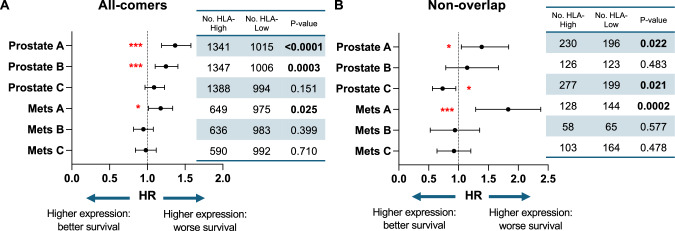
Overall survival according to HLA class I expression. Forest plots showing hazard ratios with 95% confidence intervals of multivariate analysis of overall survival (OS) stratified by HLA expression level **A** and non-overlap HLA expression **B**.

Last, we investigated the effects of HLA expression on patient outcomes. OS was compared between HLA-high and HLA-low groups, which were further stratified by biopsy sites (primary versus metastatic). Multivariate analyses were conducted, adjusted for age, race, histology, *TP53*, TMB, and MSI statuses. Worse OS was seen with HLA-A or HLA-B–high status from prostate biopsies (HR = 1.37, *p* < 0.0001; HR = 1.25, *p* = 0.0003) or HLA-A–high status from metastatic tumors (HR = 1.17, *p* = 0.025) (Fig. 5A). HLA-C expression status had no prognostic value among patients with either prostate or metastatic biopsies. Given that the majority of PC exhibited concordant up-and down-regulation of all three HLA loci (Fig. 1D), to evaluate the specific effect of each locus, we focused on the smaller subset of tumors in which only one class I HLA was changed (non-overlap groups). In this subset analysis, elevated HLA-A status alone from primary or metastatic biopsies was still associated with worse OS (HR 1.39, *p* = 0.022; HR 1.78, *p* = 0.0002). No significant findings were associated with HLA-B, whereas improved OS was seen in HLA-C–high prostate biopsies (HR 0.73, *p* = 0.021) (Fig. 5B). Altogether, the status of HLA-A was prognostic when considering OS.

### Racial Differences in HLA-Associated Survival and Underlying Genomic Alterations

Given the racial differences in HLA expression distribution (Fig. 1E), we performed univariate OS analyses stratified by race, as sample sizes were limited. In the Caucasian cohort, OS patterns were similar to the overall cohort, whereas the African cohort showed a trend toward improved survival with higher HLA expression, particularly in the HLA-A allele group, opposite to the overall trend (Supplementary Fig. 4A). To assess potential confounders, we compared known prognostic genomic alterations in prostate cancer [43] between racial groups within each HLA expression subgroup. African patients had significantly lower rates of *TMPRSS2* fusion and *TP53* alterations than Caucasian patients, while rates of *AR* mutations, *PTEN* loss, and *SMAD4* loss were comparable (Supplementary Fig. 4B). These genomic differences, along with a known HLA genotype heterogeneity among races [18], may partly explain the reversed OS trend in the African subgroup.

## Discussion

This study evaluated HLA class I levels in 8,040 PC tumors from the clinicogenomics database. High HLA expression samples exhibited distinct genomic and transcriptomic features as compared to low HLA samples. Tumors with high HLA expression had more mutations in tumor suppressors, while *AR* and *FOXA1* alterations were higher in low HLA groups. High HLA tumors showed unique gene expression patterns, including increased EMT and TGFβ pathways. Unsurprisingly, high HLA expression correlated with more immune infiltrates, checkpoint molecules, and cytokine pathways. However, despite indications of immune activation, high HLA was mostly linked to worse overall survival in PC patients. These findings underscore the complexities of measuring outcomes in PC and suggest interactions between HLA class I genes and other molecular and immunologic features in PC.

Our findings, in which PC cases have lower HLA expression compared to 66 other malignancies, align with prior evaluations of HLA in smaller studies of PC patients [16, 44]. These findings hint at the developmental process of tumors in the prostate gland, an environment without robust antitumor immunity. However, further decreased HLA expression in metastatic samples suggests that the progression towards metastasis outside of the prostate gland requires additional downregulation of immune cell interactions. Interestingly, African American patients are a subset with an increased risk of de novo metastatic disease [45, 46], and we found that a greater proportion of their tumors were lower in HLA expression. As this finding is based on statistical associations, it requires orthogonal approaches for further validation in these populations.

We examined genomic alterations in patients related to HLA expression and found pathogenic changes in *AR* and *FOXA1* linked to low expression of HLA. This aligns with PC cell line screens showing that increased AR signaling leads to reduced HLA expression [16]. Since *AR* alterations are selected in metastatic tumors [30, 31], this partially helps explain their lower HLA class I expression. Additionally, HLA-high tumors showed higher levels of AR cofactors (*HOXB13* and *FOXA1*) and enrichment of the Hallmark androgen response. Intriguingly, tumor suppressor alterations were associated with the HLA-high group. Research has shown that *TP53* and *PTEN* mutations yield neoantigens that activate TIL responses in PC [47–49]. Overall, while the independent effects in metastatic prostate tumors require further investigation, alterations in tumor suppressors and AR signaling distinctly affect HLA expression.

In HLA-high patients, we identified that *PD*-*L1* and *CTLA4* are important components of immune checkpoints that are frequently upregulated in patients with robust antitumor immunity [50]. However, when considering immune cell subsets, increased HLA expression was associated with immune cells that have pro- (M2 macrophages) or anti-tumor (NK cells, T-cells) functions [51]. Note that prior clinical studies revealed upregulation of CD8 + T cells and Tregs following ADT [52, 53]. Here, we report that PCs with a high expression of HLA-A and HLA-B tended to have higher rates of dMMR, MSI-H, and TMB-H status and, importantly, elevated TIL, all indicators for the use of pembrolizumab. In conjunction with recent studies of pembrolizumab modulation through AR signaling activity [16], it is possible that HLA expression has value in predicting ICI sensitivity, which would expand the number of eligible patients. This rationale is further supported by studies that have indicated that inadequate neoantigen presentation by HLA class I expression accounts for ICI unresponsiveness in tumors with high TMB [54–56]. However, to implement these concepts in practice, a review of HLA expression and immune cell infiltrate status must be determined through clinical trials [13, 57].

We are the first to report poor prognostic trends based on HLA class I expression in PC. Increased HLA expression is associated with improved outcomes in certain malignancies, such as colorectal, ovarian, and triple-negative breast cancer [58–63]. Here, we confirm in a large and diverse cohort that increased HLA-A and HLA-B in PC is associated with shorter OS. This poor prognosis seems counterintuitive to the increased immune cell engagement seen in these samples, which would, in theory, predict a good prognosis. However, the overall poor prognosis might be due to the increased alteration frequency of tumor suppressor genes. In PCs, these aberrant events may drive HLA class I upregulation through an adaptive (compensatory) response. Relevant to patient management, we could not determine if HLA levels were predictive of response to ICI, as the small ICI-treated sample size (*n* = 114) prevented a well-powered analysis.

### Limitations

Our clinical database is the largest cohort known to examine HLA class I expression in PC. However, this study has certain limitations. Clinical characteristics in our cohort were limited; for example, the samples do not contain cancer staging or Gleason grading data. Additionally, our database does not include germline and ploidy status of the HLA alleles, which may act as confounders when examining HLA homozygosity. Further, our TME analysis relied on quanTIseq analysis of WTS data rather than a direct method of immunohistochemistry staining or flow cytometry. Given the above limitations, data interpretation must be approached with caution. Our study lacks experimental validation to confirm mechanistic drivers of HLA expression. However, given the limited availability of preclinical models that recapitulate the immune system of prostate cancer, we believe that analysis of human specimens offers a necessary foundation for future investigations.

## Conclusions

In summary, our study explores the pattern of HLA class I expression in PC, in which we found significant associations with somatic genetic features, immune activity, and clinical outcomes. Due to these findings, we propose that it may be relevant to assess HLA class I expression levels through molecular diagnostic tests, such as that from Caris Life Sciences. These may have value as a secondary evaluation for the use of current or future forms of ICI. The many genomic and molecular associations with HLA status reported here require subsequent research through laboratory studies, in which further studies may elucidate refined mechanisms of HLA regulation in PCs.

## Supplementary information

Supplementary Method, Figures, and Tables

Supplementary Table 6

Supplementary Table 7

Supplementary Table 8

Supplementary Table 9

## Data Availability

The principal investigator of this study has full access to all the data and takes responsibility for its integrity and accuracy. The data is not publicly available due to the data usage agreement between the study team’s facilities. When possible, derived data supporting the findings of this study have been made available within the paper and its Supplementary Figures/Tables. Other data can be acquired through a letter of intent to Caris Life Sciences (https://www.carislifesciences.com/letter-of-intent/). Additional inquiries can be sent to Andrew Elliott at aelliott@carisls.com.
