# Supplementary material for: HLA class I expression shapes the tumor immune microenvironment and influences prognosis in prostate cancer
Source: Prostate Cancer Prostatic Dis. 2025 Oct 25;29(2):425–34. doi: 10.1038/s41391-025-01045-9 (PMC12980528; doi:10.1038/s41391-025-01045-9)
Supplement: Supplementary file 1 — Supplementary Method, Figures, and Tables [file 41391_2025_1045_MOESM1_ESM.pdf]

Supplementary Methods

Next-Generation Sequencing of the DNA

NGS was performed on genomic DNA isolated from formalin-fixed paraffin-embedded (FFPE) tumor samples using the NextSeq or NovaSeq 6000 platforms (Illumina, Inc., San Diego, CA). For NextSeq sequenced tumors, a custom-designed SureSelect XT assay was used to enrich 592 whole-gene targets (Agilent Technologies, Santa Clara, CA). For NovaSeq sequenced tumors, more than 700 clinically relevant genes at high coverage and high read-depth were used, along with another panel designed to enrich for an additional >20,000 genes at lower depth. All variants were detected with > 99% confidence based on allele frequency and amplicon coverage, with an average sequencing depth of coverage of > 500 and an analytic sensitivity of 5%. Prior to molecular testing, tumor enrichment was achieved by harvesting targeted tissue using manual microdissection techniques. Genetic variants identified were interpreted by board-certified molecular geneticists and categorized as 'pathogenic,' 'likely pathogenic,' 'variant of unknown significance,' 'likely benign,' or 'benign,' according to the American College of Medical Genetics and Genomics (ACMG) standards. When assessing mutation frequencies of individual genes, 'pathogenic' and 'likely pathogenic' were counted as mutations. The copy number alteration (CNA) of each exon is determined by calculating the average depth of the sample along with the sequencing depth of each exon and comparing this calculated result to a pre-calibrated value.

Copy Number Variation Analysis

To estimate gene amplification in the CNV analysis, the copy number of each exon was determined by normalizing the sequencing depth of each exon divided by the average sequencing depth of the sample and comparing it to the pre-calibrated mean of normalized values in the training data (the mean values were re-calibrated every 60 days with up to 10,000 samples). If all exons (excluding those with known insufficient coverage) in the gene of interest had an average of ≥3 copies and the average copy number of the entire gene was ≥6 copies, the gene result was reported as amplified. If an average of ≥4, but <6 copies of a gene were detected, or if the average copy number of the gene is ≥6 copies but contains exons with an average of <3 copies, the gene result was reported as intermediate. If an average of <4 copies of a gene were detected, the result was reported as not amplified.

From WES data, gene deletion was determined using open-source software CNVkit[1] (RRID:SCR_021917) to normalize read depths among on- and off-target sites for a given sample to a constructed reference. A gene was determined to be deleted if the copy number was between 0.1-1.3; intermediate loss was determined to be 1.3-1.9; not deleted was determined to be >1.9, and indeterminate was determined to be ≤0.1. Suppose the gene was not reported as amplified, and any exon (excluding the aforementioned low coverage regions) tested for the CNV call for a gene had average depth <100x. In that case, the CNV calling result for the gene was indeterminate.

RNA expression Sequencing

FFPE specimens underwent pathology review to measure percent tumor content and tumor size; a minimum of 10% of tumor content in the area for microdissection was required to enable enrichment and extraction of tumor-specific RNA. A Qiagen RNA FFPE tissue extraction kit was used for extraction, and the RNA quality and quantity were determined using the Agilent TapeStation. Biotinylated RNA baits were hybridized to the synthesized and purified cDNA targets, and the bait-target complexes were amplified in a post-capture PCR reaction. The Illumina NovaSeq 6500 was

used to sequence the whole transcriptome from patients to an average of 60M reads. Raw data was demultiplexed by Illumina Dragen BioIT accelerator, trimmed, counted, PCR-duplicates removed, and aligned to human reference genome hg19 by STAR aligner. For transcription counting, transcripts per million values were generated using the Salmon expression pipeline.

Genomic Loss of Heterozygosity Analysis

Genomic Loss of Heterozygosity (LOH) was calculated by splitting the 22 autosomal chromosomes into 552 segments and the LOH of single nucleotide polymorphisms (SNPs) within each segment. Caris WES data consist of approximately 250k SNPs spread across the genome. SNP alleles with frequencies skewed towards 0 or 100% indicate LOH (heterozygous SNP alleles have a frequency of 50%). In this assay, a segment is determined to have LOH if the average SNP variant frequency is skewed more than ± 15% from the heterozygous frequency of 50% (p-value < 0.02 after correction vs. a negative control). The final call of genomic LOH is based on the percentage of all 552 segments with observed LOH (High ≥ 16%, Low < 16%; if fewer than 3,000 SNPs can be read, the test is reported as Indeterminate). A normal epithelial ovarian genome (NA12878) that has no non-polymorphic variants, gene fusions, or other cancer hallmarks is used as a negative control. Segment sizes range from 2-6 Mb, depending on segment proximity to the centromeres or telomeres. 99% of segments are at least 5Mb. Segments excluded from the calculation of genomic LOH include those spanning ≥ 90% of a whole chromosome or chromosome arm and segments that are not covered by the SNP backbone and the WES panel. The 250k SNPs consist of 200K from exonic regions and 50K from intronic regions, with a minimum of 17 SNPs per Mb of genome sequence.

MSI/MMR status

A combination of multiple test platforms was used to determine the MSI or MMR status of the tumors profiled, including IHC (MLH1, M1 antibody; MSH2, G2191129 antibody; MSH6, 44 anti-body; and PMS2, EPR3947 antibody (Ventana Medical Systems, Inc., Tucson, AZ), fragment analysis (FA, Promega, Madison, WI) and NGS (>2800 target microsatellite loci were examined and compared to the reference genome hg19 from the University of California, Santa Cruz (UCSC) Genome Browser database). The three platforms generated highly concordant results, as previously reported [2] , and in the rare cases of discordant results, the MSI or MMR status of the tumor was determined in the order of IHC, FA, and NGS.

Microsatellite instability (MSI)-WES

Microsatellite instability by NGS was examined by the direct analysis of 2,810 known homopolymers through pentapolymer target microsatellite regions sequenced in the WES gene panel and compared to the reference genome hg38 from the UCSC Genome Browser database. The number of microsatellite loci that were altered by somatic insertion or deletion was counted for each sample. Only insertions or deletions that result in increased or decreased the number of tandem repeats were considered. Genomic variants in the microsatellite loci were detected using the same depth and frequency criteria as used for mutation detection. The threshold for MSI high (MSI-H) by NGS was determined to be 116 or more loci with insertions or deletions; equivocal to be 113-115 while stable to be 112 or less.

Publicly available datasets

We investigated two of the largest, publicly available datasets that provide DNA and RNA data[3]: the TCGA Firehose Legacy cohort[4], comprising 501 primary prostate adenocarcinoma cases, and the

SU2C/PCF cohort[5], which includes 429 metastatic prostate adenocarcinoma cases from the cBioPortal. We stratified these cohorts into HLA-high and HLA-low groups for comparison, using mRNA quartile cut points in the TCGA cohorts (approximately 125 samples in each group) and mRNA median cut points in the SU2C cohorts (around 133 samples per group), given the small sample size. Genomic alterations were defined as missense, in frame, truncating, and other mutations, excluding fusions and copy number alterations.

References

1 Talevich E, Shain AH, Botton T, Bastian BC. CNVkit: Genome-Wide Copy Number Detection and Visualization from Targeted DNA Sequencing. *PLOS Comput Biol* 2016; **12**: e1004873.

2 Vanderwalde A, Spetzler D, Xiao N, Gatalica Z, Marshall J. Microsatellite instability status determined by next-generation sequencing and compared with PD-L1 and tumor mutational burden in 11,348 patients. *Cancer Med* 2018; **7**: 746–756.

3 Cerami E, Gao J, Dogrusoz U, Gross BE, Sumer SO, Aksoy BA *et al.* The cBio cancer genomics portal: an open platform for exploring multidimensional cancer genomics data. *Cancer Discov* 2012; **2**: 401–404.

4 Broad Institute TCGA Genome Data Analysis Center. TCGA Firehose Prostate Adenocarcinoma VERSION 2016. doi:doi:10.7908/C11G0KM9.

5 Abida W, Cyrta J, Heller G, Prandi D, Armenia J, Coleman I *et al.* Genomic correlates of clinical outcome in advanced prostate cancer. *Proc Natl Acad Sci U S A* 2019; **116**: 11428–11436.

Supplementary Figures

Supplementary Figure. 1 – Pathogenic alterations in the TCGA and SU2C cohorts

Bar graphs showing percentages of pathogenic alterations in *AR, SPOP, PTEN, RB1, and CDK12* genes in the TCGA (A) and SU2C (B) cohorts.

Supplementary Figure. 2 – Differential gene expressions

Volcano plots showing differential gene expression (DGE) between PC with HLA-High vs HLA-Low status. Androgen receptor (AR), Cell surface protein (CSP), human leukocyte antigen (HLA), Immunoglobulin (IG), Immune target (IM), and Neuroendocrine (NE) related genes are highlighted. Significant genes (light grey) are those with absolute(log2FC) of >1 and a p-value of <0.01.

Supplementary Figure. 3 – Differential gene expressions in selected pathways

Bubble plots showing differential gene expression of selected cell-surface, *AR*-related, and neuroendocrine-related genes from the Caris (A), TCGA (B), and SU2C (C) cohorts.

Supplementary Figure. 4 – Racial difference in HLA-Associated Survival and Underlying Genomic Alterations

Forest plots showing hazard ratios with 95% confidence intervals of univariate analysis of OS stratified by HLA expression level in all races, Caucasian, and African populations(A). Bar graphs showing percentages of selected alterations in White/Caucasian (W) and Black/African (B) populations (B).

Supplementary Figure 1

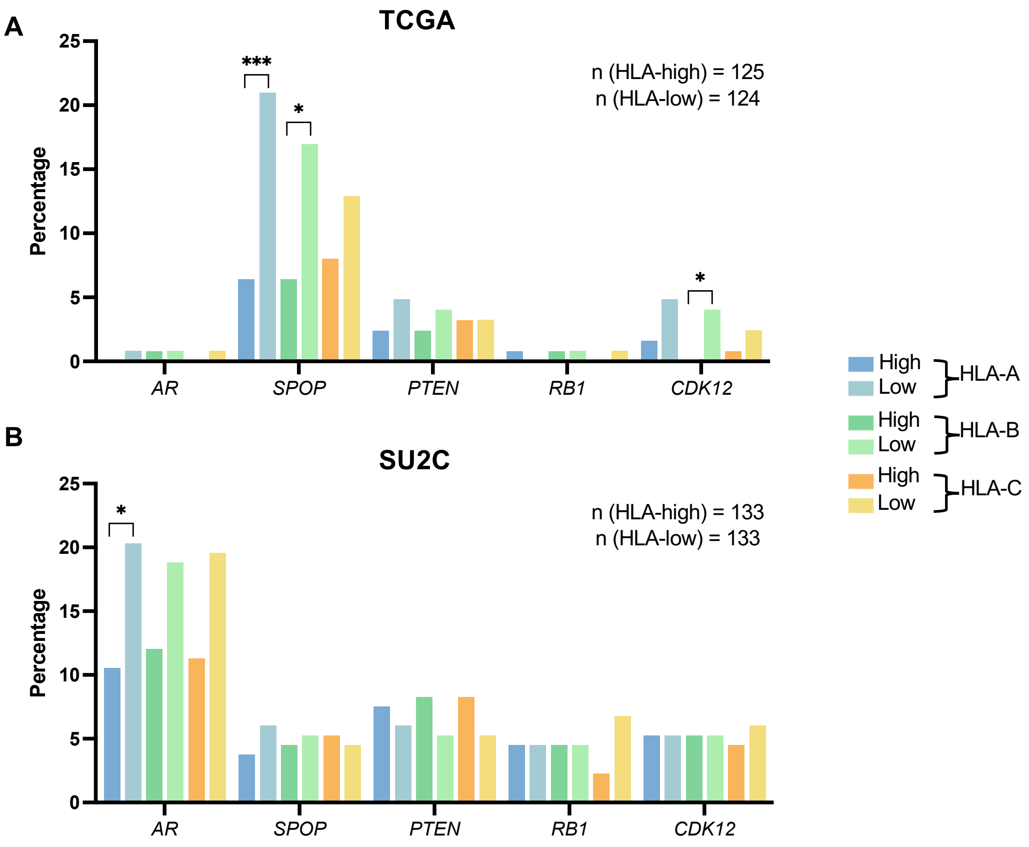

Supplementary Figure 2

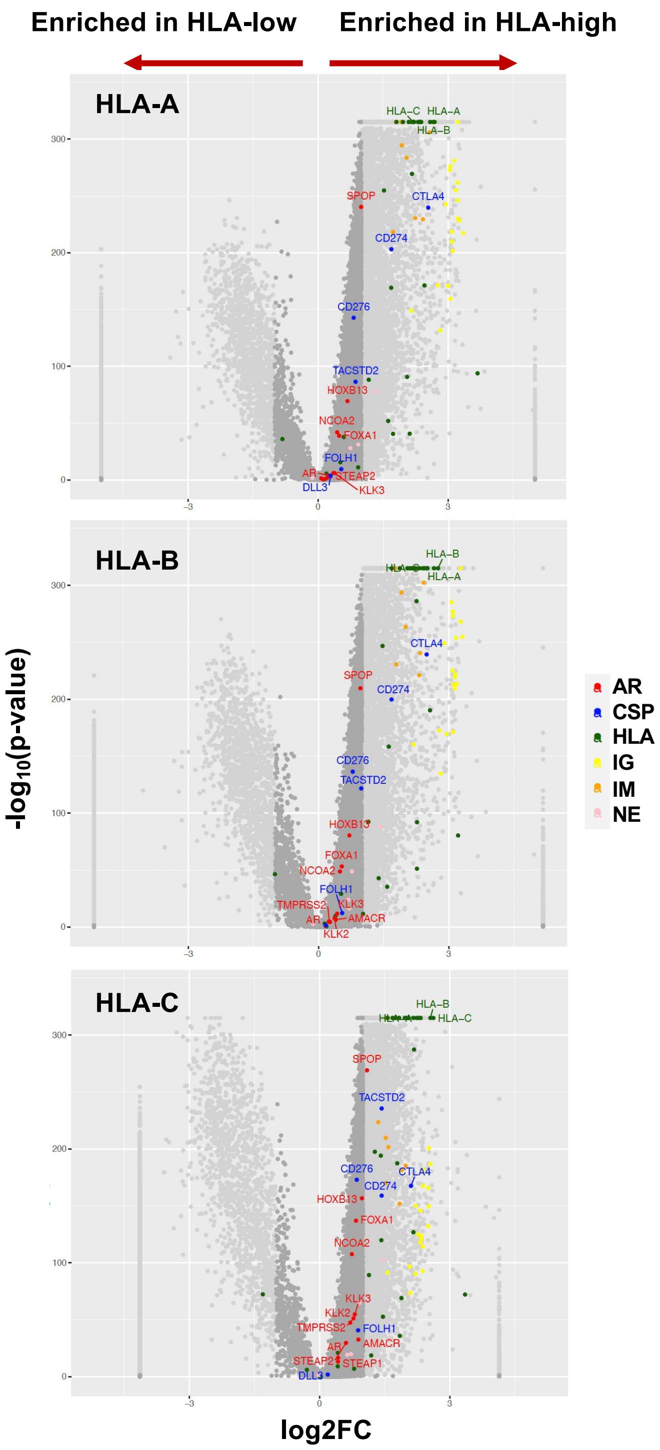

Supplementary Figure 3

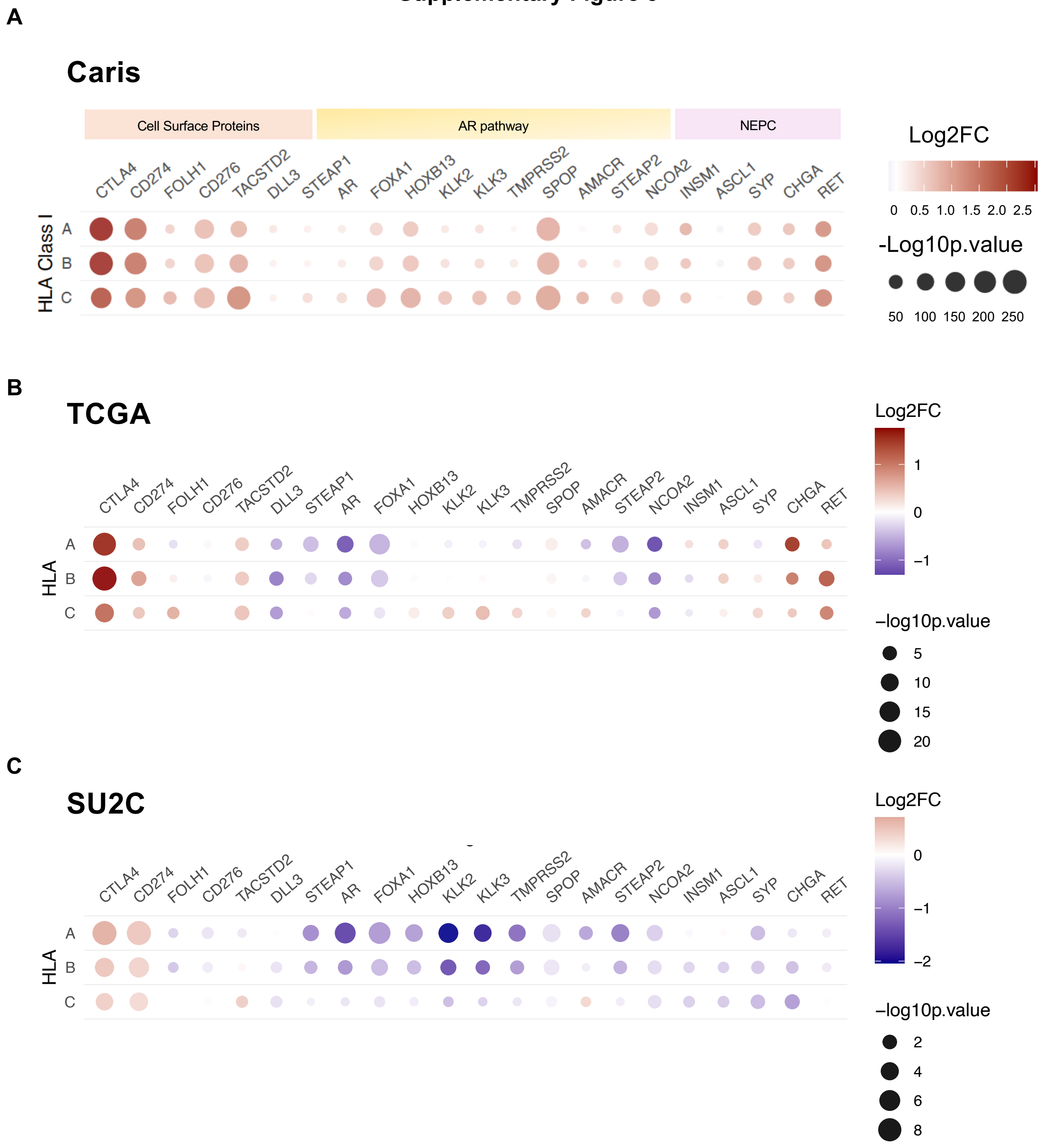

Supplementary Figure 4

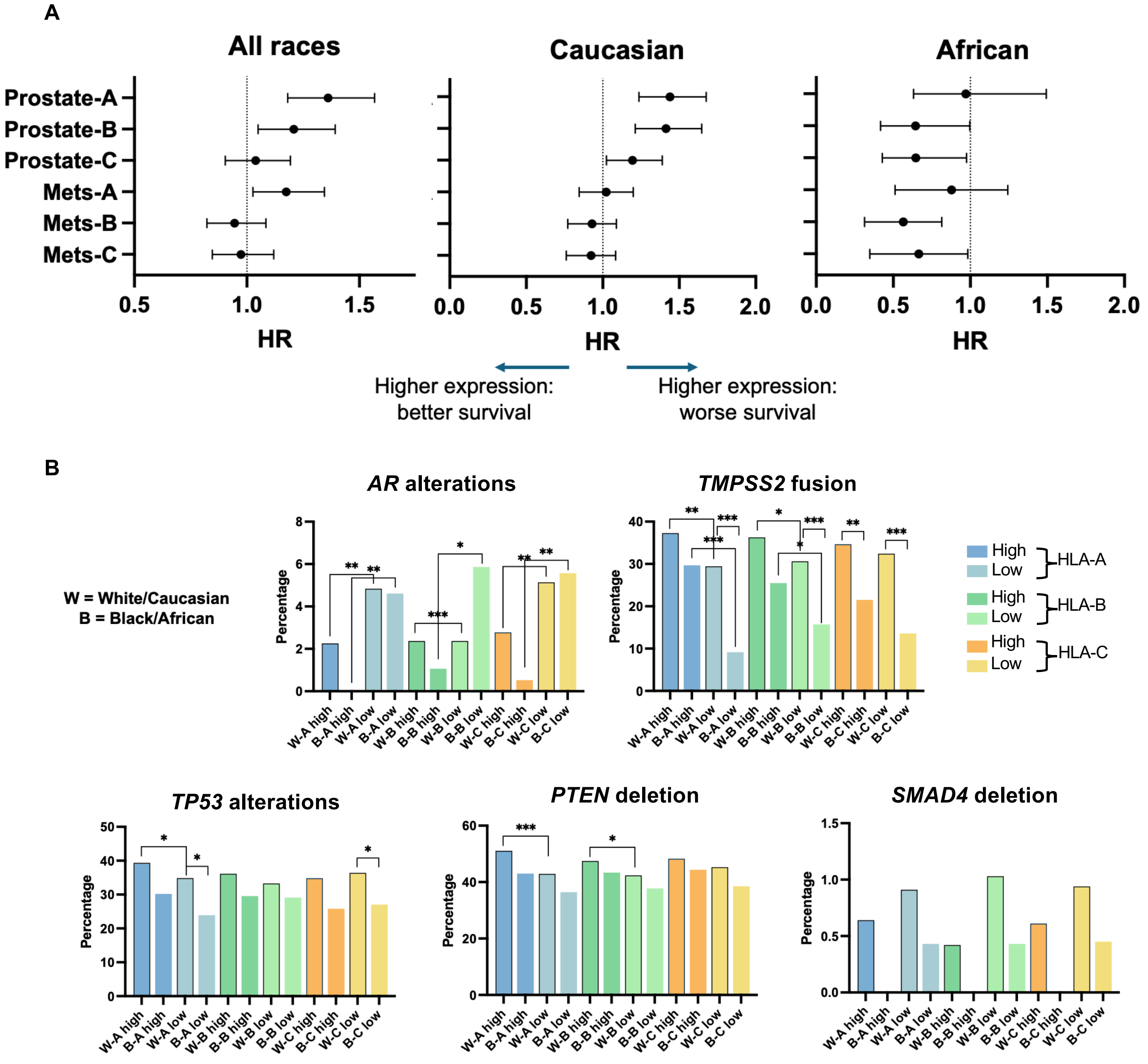

Supplementary Tables

Supplementary Table 1 - Review of HLA-related prostate cancer studies

Supplementary Table 2 - Patient demographics

Supplementary Table 3 - Patient demographics by HLA expression

Supplementary Table 4 - Cancer abbreviations for Figure 1A

Supplementary Table 5 - Race distribution for Figure 1E

Supplementary Table 6 - Median WTS expression of selected genes

Supplementary Table 7 -HLA-A volcano plot raw data

Supplementary Table 8 -HLA-B volcano plot raw data

Supplementary Table 9 -HLA-C volcano plot raw data

* *Supplementary Table 6-9 are provided in the excel format.*

Supplementary Table 1 - Review of HLA-related prostate cancer studies

Study	***Cohort Size***	***Main findings***
Likasitwatanakul, et al., 2025[1]	- 5,054 prostate and 2,986 metastatic prostate cancer tissues that had clinical-grade tumor profiling at Caris Life Science<br>- External validation with TCGA and SU2C cohorts.<br>- Data include WGS, WES, HLA genotyping, etc.	- Analysis of individual HLA – A, B, C separately<br>- High HLA class I was strongly associated with low *FOXA1* but high *TP53* alterations.<br>- High HLA class I was associated with high immune activity<br>- Worse overall survival was observed in patients with high HLA-A expression.
Rodems, et al., 2022[2]	Analyzed 2 publicly available dataset<br>1. TCGA 497 primary prostate adenocarcinoma<br>- Data include WGS, WTS, DNA methylation, ATAC-seq in a portion of data<br>2. 131 primaries and 19 metastases prostate tumors from Taylor et al, 2010[3]<br>- Data include 138 cancer-related genes DNA sequencing and microarray mRNA	- Loss of HLA-I was associated with suppressive chromatin state e.g. DNA methylation, H3K27M3<br>- Experimental confirmation with LNCaP cell and DNMT and HDAC inhibitors
Carretero, et al., 2015[4]	- 42 cryopreserved primary prostate tumors and 12 prostate benign hyperplasia samples after radical prostatectomy<br>- Data includes HLA class I immunohistochemistry, HLA genotyping, B2M mutations, q-PCR of specific genes	- Immunohistochemistry analysis to quantify HLA loss<br>- 50% of tumors showed a total loss of HLA-I and was associated with incidence of tumor relapse and perineural invasion.<br>- Samples without total loss showed locus and allelic loss.<br>- LOH of chromosome 6 in 32% of samples.
Stokidis, et al., 2023[5]<br>Stokidis, et al., 2020[6]	- Retrospective case review of 146 local and 58 de novo metastatic prostate cancer<br>- Blood for HLA typing, clinical outcomes	- HLA-A*02:01 and HLA-A*24:02 are independent prognosticators for early biochemical recurrence, castrate resistance and overall survival

Citation

1 Likasitwatanakul P, Besonen C, Sadeghipour N, Wu S, Elliott A, Arafa A *et al.* Effect of HLA class I expression on the tumor immune microenvironment and prognosis in prostate cancer. *J Clin Oncol* 2025; **43**: 5044–5044.

2 Rodems TS, Heninger E, Stahlfeld CN, Gilsdorf CS, Carlson KN, Kircher MR *et al.* Reversible epigenetic alterations regulate class I HLA loss in prostate cancer. *Commun Biol* 2022; **5**: 1–16.

3 Taylor BS, Schultz N, Hieronymus H, Gopalan A, Xiao Y, Carver BS *et al.* Integrative genomic profiling of human prostate cancer. *Cancer Cell* 2010; **18**: 11.

4 Carretero FJ, Campo AB del, Flores-Martín JF, Mendez R, García-Lopez C, Cozar JM *et al.* Frequent HLA class I alterations in human prostate cancer: molecular mechanisms and clinical relevance. *Cancer Immunol Immunother CII* 2015; **65**: 47.

5 Stokidis S, Baxevanis CN, Fortis SP. The Prognostic Significance of Selected HLA Alleles on Prostate Cancer Outcome. *Int J Mol Sci* 2023; **24**: 14454.

6 Stokidis S, Fortis SP, Kogionou P, Anagnostou T, Perez SA, Baxevanis CN. HLA Class I Allele Expression and Clinical Outcome in De Novo Metastatic Prostate Cancer. *Cancers* 2020; **12**: 1623.

Supplementary Table 2 - Patient demographics

		**Prostate biopsy**		**Metastatic biopsy**		**p-value**
**PCs with HLA expression available**		**N=5054**	**%**	**N=2986**	**%**	
**PCs with HLA genotype available**		1771	35	870	29.1	**<0.001**
**Mean age (years)**		67		70		
**Race**	White	3249	64.3	1854	62.1	**0.049**
	Black/African	617	12.2	451	15.1	**<0.001**
	Asian	117	2.3	62	2.1	0.532
	Other	127	2.5	92	3.1	0.137
	Unknown	471	9.3	304	10.2	0.211
**Histology**	Adenocarcinoma	4949	97.9	2829	94.7	**<0.001**
	Neuroendocrine	56	1.1	107	3.6	**<0.001**
**Treatment**	Radiation	2293	45.4	1467	49.1	**0.001**
	1st generation ADT	3031	60	863	28.9	**<0.001**
	2nd generation ADT	2223	44	762	25.5	**<0.001**
	Taxane	1220	24.1	782	26.2	**0.043**
	Immune checkpoint inhibitor	153	3	152	5.1	**<0.001**

Supplementary Table 3 - Patient demographics by HLA expression

		**HLA-High**		**HLA-Low**		**p-value**
		N=2914	%	N=2847	%	
**HLA types**	HLA-A	2010	68.98	2007	70.5	0.218
	HLA-B	2011	69.01	2005	70.43	0.251
	HLA-C	2009	68.94	2005	70.43	0.229
	Mean age	68		68		
**Race**	White	1910	65.55	1681	59.04	**3.66E-07**
	Black/African	368	12.63	539	18.93	**5.21E-11**
	Asian	74	2.54	84	2.95	0.375
	Other	113	3.88	102	3.58	0.578
	Unknown	168	5.77	184	6.46	0.272
**Specimen sites**	Prostate	1985	68.12	1483	52.09	**1.57E-35**
	Metastatic	929	31.88	1364	47.91	**1.57E-35**
**Histology**	Adenocarcinoma	2837	97.36	2794	98.14	0.051
	Neuroendocrine	71	2.44	45	1.58	**0.024**
**Treatment**	Radiation	1338	45.92	1345	47.24	0.316
	1st gen ADT	1388	47.63	1349	47.38	0.854
	2nd gen ADT	1057	36.27	1030	36.18	0.956
	Taxane	678	23.27	763	26.8	**0.002**
	ICI	112	3.84	99	3.48	0.483

Supplementary Table 4 - Cancer abbreviations for Figure 1A

ANCA	Anal carcinoma	MELA	Melanoma
APCA	Appendiceal cancer	MENI	Meningioma
BCSC	Basal Cell Skin cancer	MCC	Merkel Cell Carcinoma MCC
BLCA	Bladder cancer	MIS	Misclassified Cases
BONE	Bone cancer	MM	Multiple Myeloma
BRCA	Breast carcinoma	NEU	Neuroendocrine tumours
CUP	Cancer of Unknown Primary	NDLB	Nodal Diffuse Large B-cell Lymphoma
CECA	Cervical cancer	NEOV	Non Epithelial Ovarian Cancer non-EOC
CCAE	Cholangiocarcinoma, extrahepatic	None	None of these apply
CCAG	Cholangiocarcinoma, gallbladder cancer	OV	Ovarian Surface Epithelial carcinomas
CCAI	Cholangiocarcinoma, intrahepatic	PAAD	Pancreatic Adenocarcinoma
CCAN	Cholangiocarcinoma, NOS	PECA	Penile Cancer
COAD	Colorectal Adenocarcinoma	PNST	Peripheral Nervous System Tumors
ENCA	Endometrial carcinoma	PICA	Pituitary Carcinomas
ENSA	Endometrial sarcoma	PRAD	Prostatic Adenocarcinoma
EPEN	Ependymoma	RECA	Retroperitoneal or Peritoneal carcinoma
ESCA	Esophageal and esophagogastric junction carcinoma	SLGT	Salivary Gland tumors
FGTM	Female Genital Tract Malignancy	SIM	Small Intestinal malignancies
GAAD	Gastric Adenocarcinoma	STTS	Soft Tissue tumor - Solitary Fibrous Tumor_Hemangiopericytoma
GIST	Gastrointestinal Stromal tumors GIST	STT	Soft Tissue tumors
HNCA	Head and Neck cancer	STTW	Soft Tissue tumors - WD-DDLS
HGGL	High Grade glioma	SCSC	Squamous Cell Skin cancer
KICA	Kidney cancer	TECA	Testicular cancer
LEUK	Leukemia	THYM	Thymomas and Thymic Carcinomas
LIHC	Liver Hepatocellular carcinoma	THCA	Thyroid carcinoma-anaplastic
LGGL	Low Grade glioma	THCF	Thyroid carcinoma-Follicular
LUNS	Lung Non-small Cell Lung cancer NSCLC	THCH	Thyroid carcinoma-Hurthle Cell
LUSC	Lung Small Cell Cancer SCLC	THCM	Thyroid carcinoma-Medullary
LYMP	Lymphoma	THCN	Thyroid carcinoma-NOS
MGTM	Male Genital Tract Malignancy	THCP	Thyroid carcinoma-Papillary
MHIS	Malignant Histiocytosis	USCA	Uterine Serous Carcinoma
MESO	Malignant Pleural Mesothelioma	UVM	Uveal Melanoma
MEDU	Medulloblastoma	VUCA	Vulvar Cancer Squamous Cell carcinoma

Supplementary Table 5 - Race Distribution for Figure.1E

							Fischer test (p-value)		
	HLA-A high	***HLA-A low***	***HLA-B high***	***HLA-B low***	***HLA-C high***	***HLA-C low***	***HLA A high vs low***	***HLA B high vs low***	***HLA C high vs low***
**Caucasian**	73.12	64.10	74.30	63.52	73.40	63.94	**4.38E-09**	**1.97E-12**	**6.94E-10**
**Black/African**	11.40	18.98	11.59	18.68	11.78	18.73	**1.45E-10**	**1.94E-09**	**4.94E-09**
**Asian/PI**	2.24	3.36	2.30	2.93	2.04	2.76	**0.045**	0.255	0.162
**Other**	3.05	2.82	2.73	3.20	2.75	3.36	0.697	0.437	0.293
**Unknown**	10.20	10.74	9.08	11.67	10.02	11.21	0.628	**0.011**	0.260
